# Molecular basis of transient neonatal diabetes mellitus in Japan: frequent KATP-TNDM and identification of a patient with a monoallelic INS mutation

**DOI:** 10.1186/1687-9856-2015-S1-O32

**Published:** 2015-04-28

**Authors:** Tohru Yorifuji, Yuki Hosokawa, Rie Kawakita, Yukiko Hashimoto, Azumi Sakakibara, Rika Fujimaru, Nobuyoshi Tamagawa

**Affiliations:** 1 Department of Pediatric Endocrinology and Metabolism, Children's Medical Center, Osaka City General Hospital, Japan; 2 Department of Genetic Medicine, Osaka City General Hospital, Japan; 3 Clinical Research Center, Osaka City General Hospital, Japan

## Background

It has been reported that the most common (~70%) form of transient neonatal diabetes mellitus (TNDM) is 6q24-related TNDM, followed by TNDM caused by activating mutations in the KATP channel genes (KCNJ11 and ABCC8, KATP-TNDM) which accounts for approximately 30% of TNDM. Recessive promoter mutations in the insulin (INS) gene also have been reported as rare causes of TNDM.

## Aims

To elucidate the molecular basis of TNDM in Japan.

## Methods

Nineteen Japanese patients with TNDM were analysed by methylation specific PCR of the differentially methylated region at chromosome 6q24 and by PCR amplification and direct sequencing of all exons and exon-intron boundaries of the KCNJ11, ABCC8, and INS genes.

## Results

6q24 abnormalities were identified in 7 (paternal duplication in 4, paternal uniparental disomy or epimutation in 3), mutations of ABCC8 (R1380C, R216C, V607M) in 3, KCNJ11 (E227K, R50Q, C42R) in 3, and INS (Q62X) in 1.

## Conclusion

As compared with previous reports, the frequency of KATP channel mutations were higher in this Japanese cohort. In addition, this is the first report of a monoallelic, coding sequence mutation in the INS gene (Q62X) responsible for TNDM.

